# Supplementary material for: Predicting visual function by interpreting a neuronal wiring diagram
Source: Nature. 2024 Oct 2;634(8032):113–23. doi: 10.1038/s41586-024-07953-5 (PMC11446822; doi:10.1038/s41586-024-07953-5)
Supplement: Supplementary file 6 — CRF and ERF predictions for individual TmY4 and TmY9 cells. Analogous to Supplementary Data 3, but for TmY target types. Shown are the top four monosynaptic pathways, the strongest pathway passing through each of the top ten intermediary types (ranking from Extended Data Fig. 7), and the trisynaptic pathway Tm1–TmY–Dm3–TmY (see the section entitled Prediction of spatial normalization). [file 41586_2024_7953_MOESM6_ESM.zip › DataS4/TmY9q/720575940625360572.pdf]

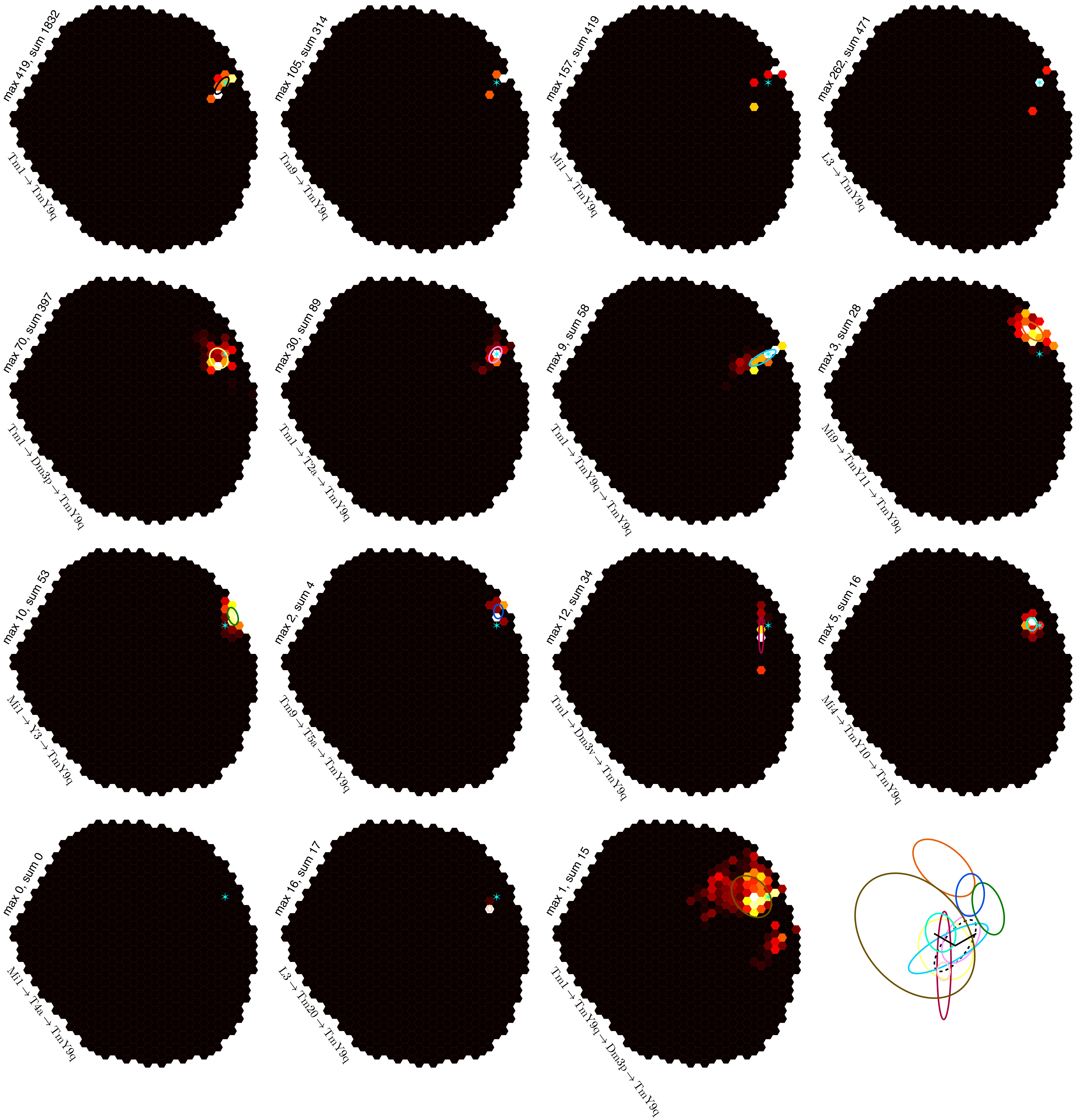
max 419, sum 1832
Tm1 → TmY9q
max 105, sum 314
Tm9 → TmY9q
max 157, sum 419
Mi1 → TmY9q
max 262, sum 471
L3 → TmY9q
max 70, sum 397
Tm1 → Dm3p → TmY9q
max 30, sum 89
Tm1 → T2a → TmY9q
max 9, sum 58
Tm1 → TmY9q → TmY9q
max 3, sum 28
Mi9 → TmY11 → TmY9q
max 10, sum 53
Mi1 → Y3 → TmY9q
max 2, sum 4
Tm9 → T5a → TmY9q
max 12, sum 34
Tm1 → Dm3v → TmY9q
max 5, sum 16
Mi4 → TmY10 → TmY9q
max 0, sum 0
Mi1 → T4a → TmY9q
max 16, sum 17
L3 → Tm20 → TmY9q
max 1, sum 15
Tm1 → TmY9q → Dm3p → TmY9q
